# Supplementary figures and images for: Reovirus uses temporospatial compartmentalization to orchestrate core versus outercapsid assembly
Source: PLoS Pathog. 2022 Sep 13;18(9):e1010641. doi: 10.1371/journal.ppat.1010641 (PMC9514668; doi:10.1371/journal.ppat.1010641)

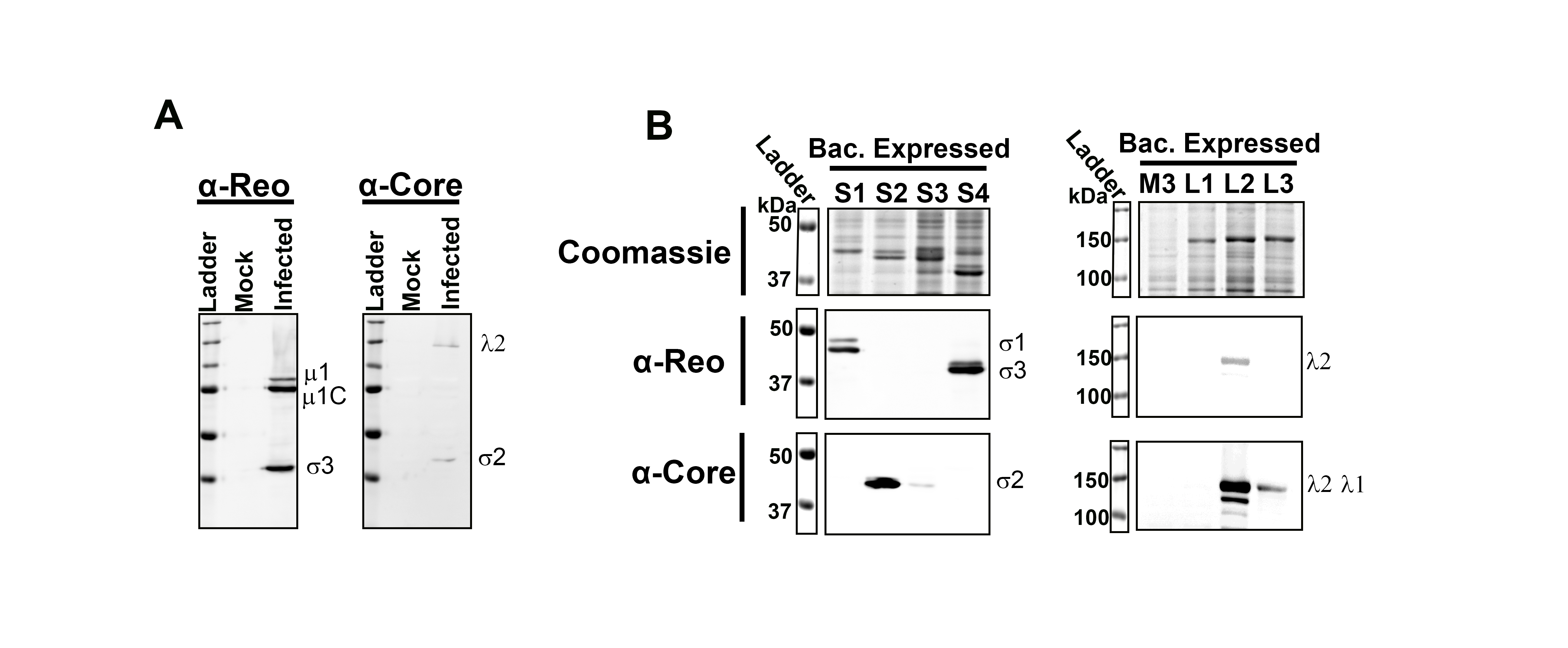

Supplement: S2 Fig — (A) L929 cells were infected with reovirus at an MOI of 3, collected and lysed at 14 hpi. Mock-infected and infected lysates were subject to SDS-PAGE and Western blot analysis using polyclonal antibodies raised against whole virus (α-Reo, left) or (α-Core, right). (B) Sf9 insect cells were infected with baculoviruses expressing reovirus proteins. Cells were lysed and proteins were analyzed by SDS-PAGE and coomassie staining (top) or Western blot (middle, α-Reo and bottom, α-Core). (TIF) [file ppat.1010641.s002.tif]

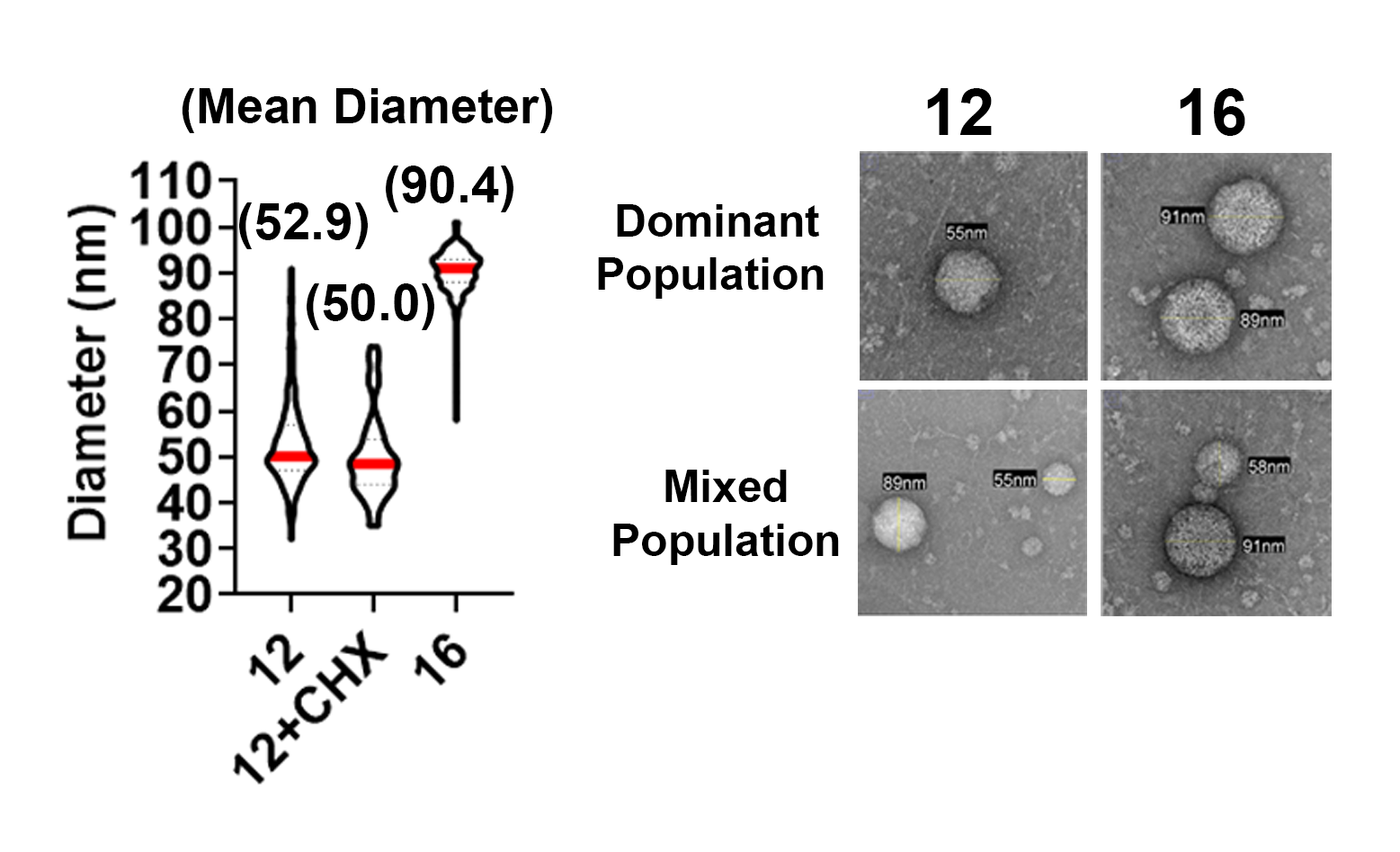

Supplement: S4 Fig — L929s were infected with reovirus at MOI 3 in presence or absence of cycloheximide (CHX). At 12 and 16hpi, whole lysates were subjected to high-speed ultracentrifugation through 1.33g/cc CsCl to pellet reovirus cores and fully-assembled viruses, and imaged via TEM. Particle diameters of 206, 42 and 443 particles captured from lysates collected at 12hpi without CHX, at 12hpi with CHX, or at 16hpi without CHX were measured using Figi ImageJ and each diameter plotted in the violin plot (Left). (Right), example micrographs from 12hpi and 16hpi show the most dominant population from the lysates (top) versus an example of micrographs showing cores and whole viruses side-by-side for comparison. (TIF) [file ppat.1010641.s004.tif]

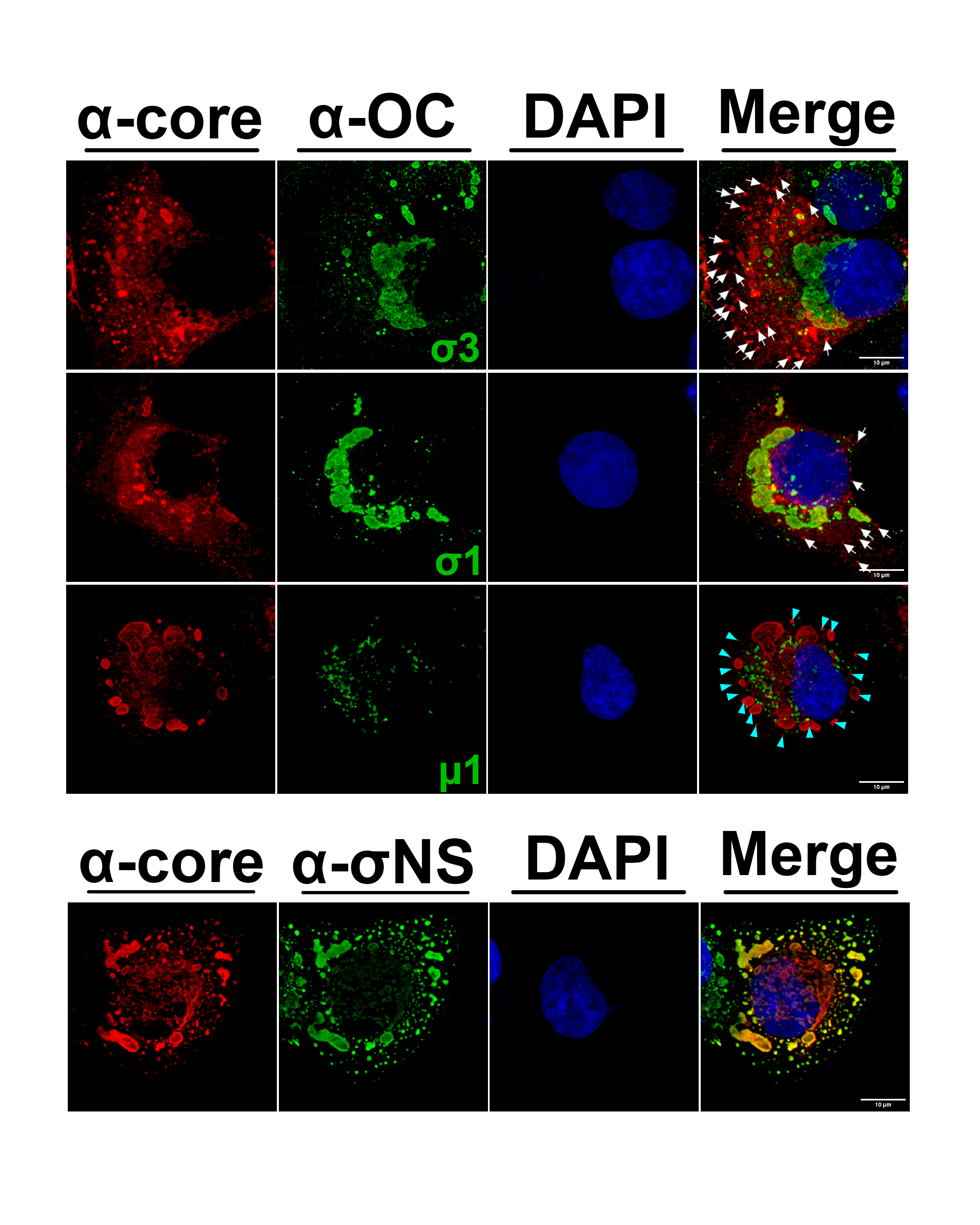

Supplement: S5 Fig — T47D cells were infected with reovirus at an MOI of 3 before fixation at 22 hpi. Immunofluorescence staining was conducted with antibodies specific to OC proteins indicated in green (monoclonal 10G10 for σ3, monoclonal G5 for σ1, or monoclonal 10F6 for μ1 as indicated) or σNS (monoclonal 2A9, bottom). The OC proteins were detected with secondary antibodies conjugated to Alexa 488 (pseudo colored green) or Alexa 647 (pseudo-colored red). Co-immunofluorescence in the same cells was conducted using polyclonal rabbit antibodies raised against reovirus cores (α-Core) detected with secondary antibodies conjugated to Alexa 647 (red). In the merged images, white arrows show example regions of core-only staining, while cyan arrows indicate regions of core-positive but μ1-negative. (TIF) [file ppat.1010641.s005.tif]

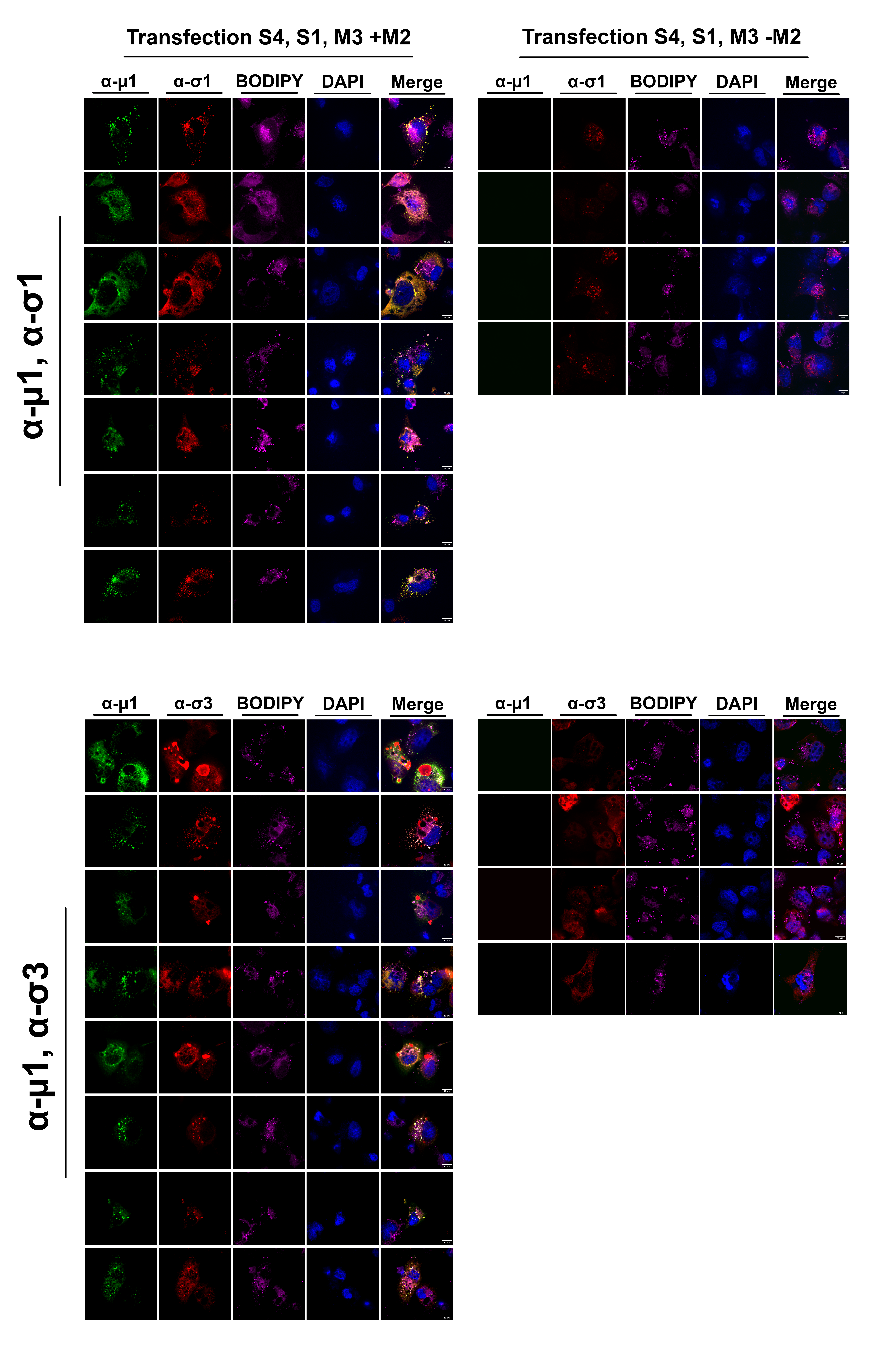

Supplement: S6 Fig — H1299 cells were transfected with S1pcDNA3 (σ1), S4pcDNA3 (σ3), and M3pcDNA3 (μNS) with (left) or without M2pcDNA3 (μ1) (right). Immunofluorescence staining was conducted with antibodies specific to OC proteins μ1 (monoclonal 10F6) and σ1 (monoclonal G5 directly labelled with AlexaFluor 647) (top) or μ1 (10F6) and σ3 (monoclonal 10C1 directly labelled with AlexaFluor 647) (bottom), BODIPY 568 for LDs, and DAPI for nuclei. μ1 was detected with secondary antibodies conjugated to AlexaFluor 488. Represented images were created from Z-stacks acquired using immunofluorescent spinning disk confocal microscopy. (TIF) [file ppat.1010641.s006.tif]

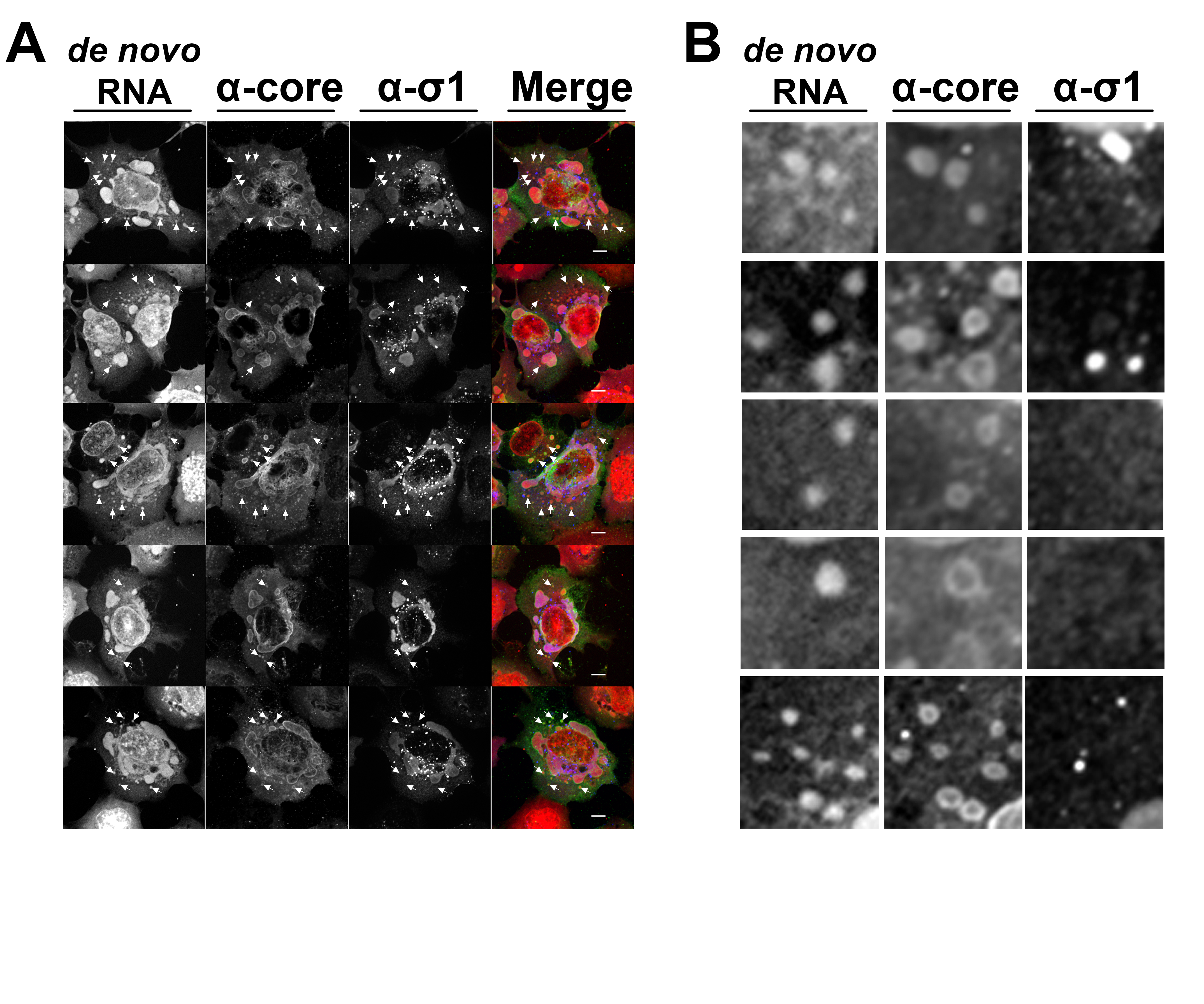

Supplement: S7 Fig — (A) H1299 cells were infected with reovirus at an MOI of 3 and at 14hpi, cells were treated with actinomycin D to reduce cell transcription. Between 15 hpi and 18 hpi, cells were stained for de novo transcribed RNA using an EZ-click RNA Labelling kit (RNA, red in merged image). Fixed cells were processed for immunofluorescence with rabbit polyclonal α-core antibodies (Alexa Fluor 488, green in merged image) and monoclonal mouse α-σ1 antibody G5 (Alexa Fluor 405, blue in merged images). Images were captured by spinning disk confocal microscopy. Scale bars represent 20μm. White arrows represent example regions positive for core and RNA staining, but negative for σ1. (B) Close-up images show examples of OC negative, core-positive foci, which are consistently also positive for de novo RNA. (TIF) [file ppat.1010641.s007.tif]

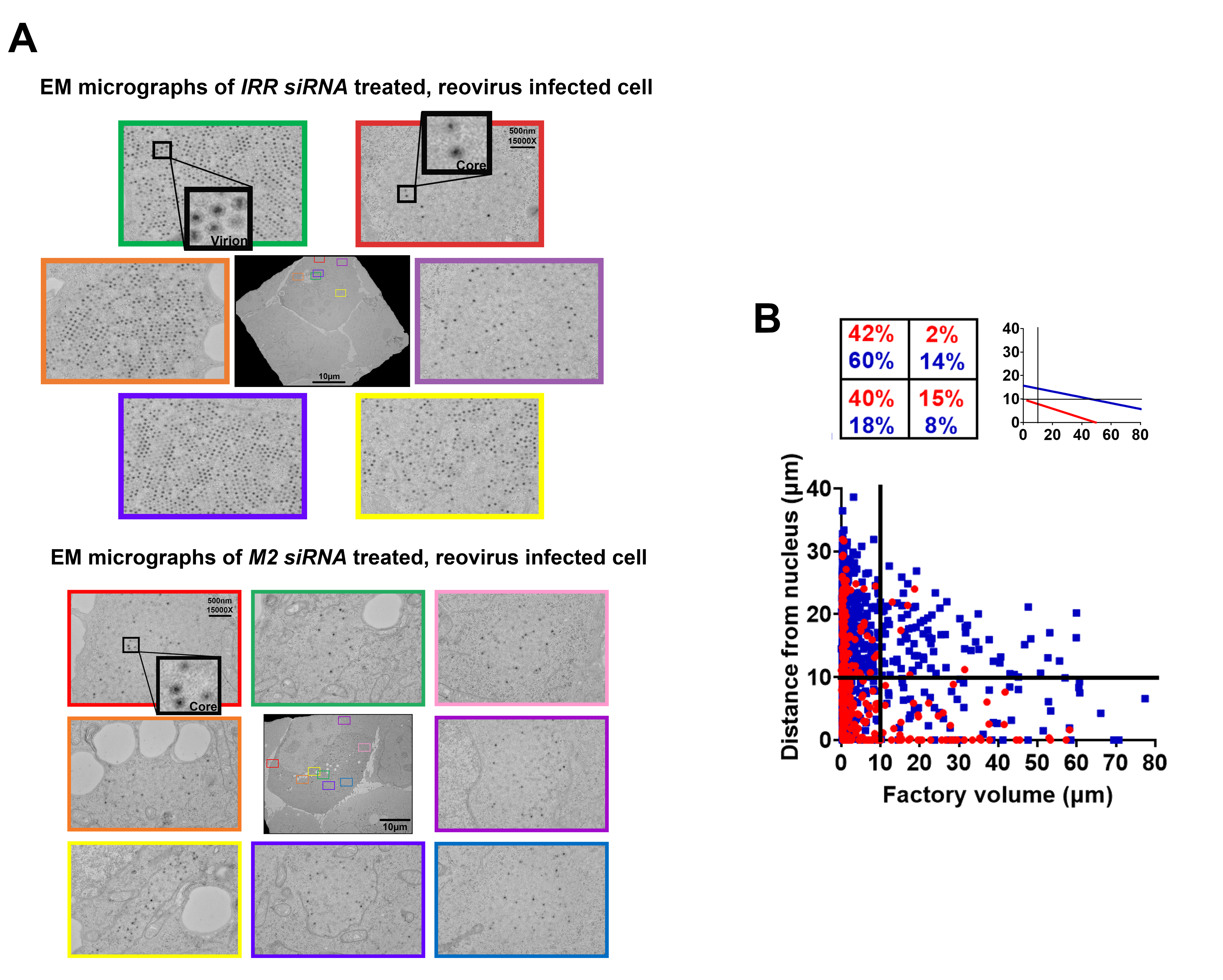

Supplement: S10 Fig — Prior to infecting H1299 cells with reovirus at an MOI of 3, cells were transfected with DsiRNAs: an irrelevant control DsiRNA (IRR) or test condition (M2 gene; μ1 reovirus outercapsid protein). (A) Cells were fixed at 17hpi and imaged by TEM. Example images from various regions around a representative irrelevant control DsiRNA (IRR, top) or M2/μ1 (bottom) DsiRNA-transfected cell show the particle composition of factories found in each, with core regions only found in IRR siRNA transfected cells. Select particles are highlighted in black boxes. (B) The volume and distance from the nucleus were measured for each factory in IRR (blue squares) versus M2/μ1 (red circles). DsiRNA-treated cells, using α-core channel to capture both core-only and core+OC shared factories. Based on the graph (same as found in Fig 9F), quadrants were established based arbitrarily at 10μm3 volume and 10μm distance from the nucleus to compare ratio of factories between IRR- and M2/μ1 DsiRNA-treated cells. (Top left) The percent of factories in each quadrant. (Top right) Linear regression analysis to depict trends in the distribution of factories. Data represents 8 images per condition and is representative of 2 independent experiments. (TIF) [file ppat.1010641.s010.tif]

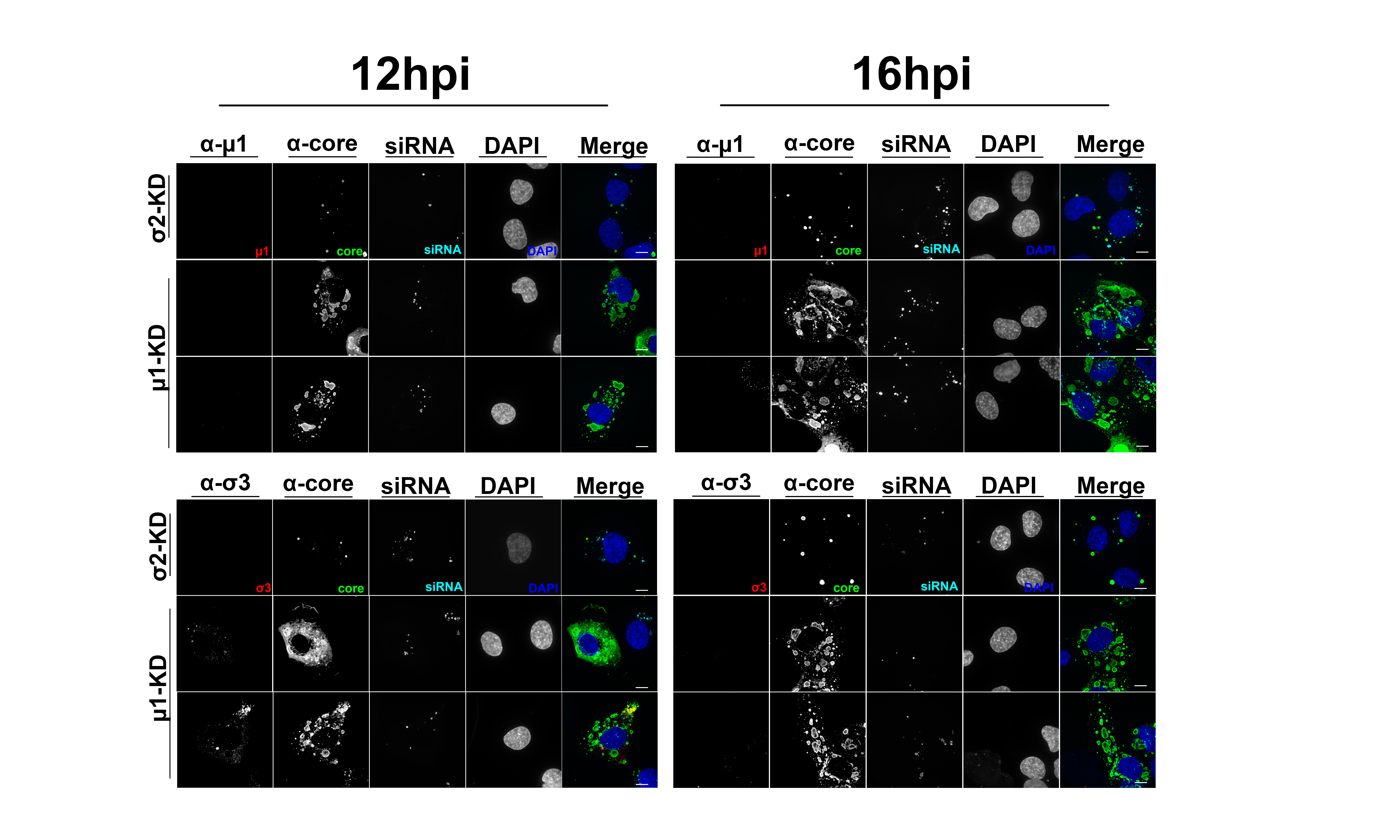

Supplement: S11 Fig — Representative images of DsiRNA-treated infected cells at 12hpi and 16hpi. Cells were immunofluorescently labelled with monoclonal mouse α- μ1 (Top, 10F6, Alexa Fluor 647, red in merged images) or monoclonal mouse α-σ3 (Bottom, 10G10, Alexa Fluor 647, red in merged images) in combination with polyclonal rabbit α-core (Alexa Fluor 488, green in merged images), Tye563 for siRNA staining (cyan), and DAPI for nuclei staining (blue). All images were acquired via immunofluorescence spinning disk confocal microscopy. (TIF) [file ppat.1010641.s011.tif]
