# Supplementary material for: Reovirus uses temporospatial compartmentalization to orchestrate core versus outercapsid assembly
Source: PLoS Pathog. 2022 Sep 13;18(9):e1010641. doi: 10.1371/journal.ppat.1010641 (PMC9514668; doi:10.1371/journal.ppat.1010641)
Supplement: S3 Fig — RNA translation efficiency measured by Kozak similarity score and the number of labelled amino acids through incorporation of S35 labelled cysteine and methionine. The relative levels of radiation per protein per virus particle are also indicated. (PDF) [file ppat.1010641.s003.pdf]

Protein Name	RNA Sequence Name	Start Sequence	Kozak Similarity Score	#Cysteines	#Methionines	Total # Labelled Amino Acids	Protein Length (amino acids)	Copies/virus particle	Radiation per virus particle
**σ1**	S1	GTCGG **ATG** G	0.57	1	8	9	455	≤36	324
**σ2**	S2	CAGTT **ATG** G	0.67	8	10	18	418	120	2160
**σNS**	S3	TCACT **ATG** G	0.81	14	22	36	366	0	0
**σ3**	S4	TCGCA **ATG** G	0.78	6	21	27	365	600	16200
**μ2**	M1	CGGTC **ATG** G	0.81	11	27	38	736	≤12	456
**μ1/ μ1C**	M2	CAAAG **ATG** G	0.80	4	18	22	708	600	13200
**μNS**	M3	TGGTC **ATG** G	0.80	12	21	33	721	0	0
**λ3**	L1	CGACA **ATG** T	0.69	16	49	65	1267	≤12	780
**λ2**	L2	GCGCG **ATG** G	0.80	15	28	43	1289	60	2580
**λ1**	L3	TCAGG **ATG** A	0.74	13	43	56	1275	120	6720
