# Supplementary material for: Reovirus uses temporospatial compartmentalization to orchestrate core versus outercapsid assembly
Source: PLoS Pathog. 2022 Sep 13;18(9):e1010641. doi: 10.1371/journal.ppat.1010641 (PMC9514668; doi:10.1371/journal.ppat.1010641)
Supplement: S12 Fig — RT-PCR reactions were executed following the Sybr Select (4472920, Invitrogen) protocol using reovirus gene-specific primers listed. (DOCX) [file ppat.1010641.s012.docx]

TABLE 1: qPCR primer sequences

| **Primer Name** | **Sequence** |
| --- | --- |
| S1RTfwd1 | TCACCCAGGGACTCGATGAT |
| S1RTrev1 | GGGCTCCGATAGAGCTTTCC |
| S1RTfwd2 | AGGGTACAGGTGAACTCCGA |
| S1RTrev2 | ATGAAAAGCGGGCTCAGTGT |
| S2RTfwd1 | ACGCTTAGTGTGGTCAGCTC |
| S2RTrev1 | TGAATCTTGGATCACGCGCT |
| S2RTfwd2 | AACATCCATGGCTGGACTGG |
| S2RTrev2 | GAGTCTGATCTGCCAACGCT |
| S3RTfwd1 | CAACTCCTGATGCGCCAATG |
| S3RTrev1 | AGCCATGCCCATGGTAAACA |
| S3RTfwd2 | ACCCTTCAGTGAGCGTGTTT |
| S3RTrev2 | TGCTGAACGAGTCCACAGTC |
| S4RTfwd1 | TTACTGCTCACTGGAAGCGC |
| S4RTrev1 | GTGAACCTCCCTCAGTACGC |
| S4RTfwd2 | TTCTAGGGCGACTACCCCAA |
| S4RTrev2 | TAGCTTGCGCACTGTCTTCA |
| M1RTfwd1 | CAACGTTGATCGTCGGCTTC |
| M1RTrev1 | GAGAGGTGCGTAGACATCCG |
| M1RTfwd2 | GATCAGCTTGTCGTAGGCGA |
| M1RTrev2 | ACTGTATGCAGCTCCACGTC |
| M2RTfwd1 | AATCAGCCTTGGTGCCCTAC |
| M2RTrev1 | CTGACAGCACACGCATCTTG |
| M2RTfwd2 | TAGTGCACTGGCACCTTCAG |
| M2RTrev2 | ATTATCTCAGGCTCAGCCGC |
| M3RTfwd1 | AAGCGCTTTGCCATAAAGCC |
| M3RTrev1 | GACACGCTGGTTGAAGCAAG |
| M3RTfwd2 | ATCCAGTCAGACCCTACCCC |
| M3RTrev2 | CTTGACATCCGCCTCCAACT |
| L1RTfwd1 | GGGATTGCGAAATCAGGTGC |
| L1RTrev1 | GCACTACCAGACGTGGTTGA |
| L1RTfwd2 | GGGAGAAATATGGAGCGGGG |
| L1RTrev2 | CCAGCTCTTCTCTCGTAGCG |
| L2RTfwd1 | TCCGTTGTTGATGGGGCTAC |
| L2RTrev1 | CATCAGATCGCCTTCGTCCA |
| L2RTfwd2 | TGCAGGCTCTAGTGGGATCT |
| L2RTrev2 | CATCAGTTCCCGCGATCGTA |
| L3RTfwd1 | CCCCGATGCTGAGAAATGGT |
| L3RTrev1 | TGCTCGATCAAACCGTCCAA |
| L3RTfwd2 | ATTACTCAGCCGGCGCTATC |
| L3RTrev2 | TGGGTAAATGGCATCAGCGT |
